# Experiences of physiotherapists audited by South African medical funding schemes

**DOI:** 10.4102/sajp.v82i1.2261

**Published:** 2026-02-13

**Authors:** Lesley Meyer, Werdie van Staden, Karien Mostert

**Affiliations:** 1 Department of Physiotherapy, Faculty of Health Sciences, University of Pretoria, Pretoria, South Africa; 2 Centre for Ethics and Philosophy of Health Sciences, Faculty of Health Sciences, University of Pretoria, Pretoria, South Africa

**Keywords:** audit, conflicts of interest, entrapment, healthcare, medical schemes, physiotherapy, private providers, Section 59.3

## Abstract

**Background:**

Forensic auditing safeguards patients’ funds by investigating billing irregularities of claims submitted to medical schemes. However, billing irregularity audits can be misguided, as indicated in a study conducted in Australia, Canada and the United States. Negative consequences were reported, including attrition, and patients funding their treatments out of pocket. Medical professionals were penalised, without forensic auditors considering alternative explanations for the alleged irregular billing patterns. Small medical practitioners, unable to defend themselves financially, closed their practices, leaving patients without medical care. In South Africa, the experiences of physiotherapists who underwent forensic audits have not been examined.

**Objectives:**

This study explored physiotherapists’ experiences of forensic auditing by medical scheme administrators.

**Method:**

An analytical qualitative study was conducted to interview 14 physiotherapists. Three focus group discussions and 11 individual interviews were conducted. A semi-structured open-ended interview guide was used, analysing the data via open and axial coding.

**Results:**

The first five themes that emerged captured the adverse experiences of physiotherapists. These were (1) ‘unfairly persecuted, judged and penalised’; (2) ‘overpowered and oppressed’; (3) ‘naively entrapped between a rock and a hard place’; (4) ‘distressed with a knife over your head’ and (5) ‘detrimental and hurtful’. In the sixth theme, ‘seeking remedies pre-emptively and preparedly’, the participants made recommendations to prevent similar unwanted experiences.

**Conclusion:**

Physiotherapists experienced significant emotional, financial and professional detriment at the hands of South African medical scheme administrators.

**Clinical Implications:**

The quality of care provided by physiotherapists is adversely affected when forensic audit-related distress occurs.

## Introduction

Forensic audits in the healthcare sector are financial investigations initiated to detect irregular billing claims submitted to healthcare funding bodies. While audits are intended to safeguard individuals’ funds, they may have serious consequences for practitioners and their patients if conducted unfairly, internationally and in South Africa. In Australia, Canada and the United States, billing irregularity audits were reported to have had detrimental effects on healthcare delivery (Meyer, Mostert & Van Staden 2024). Australia reported an out-of-pocket crisis, and small practices unable to afford legal counsel closed, leaving individuals without access to care (Faux, Adams & Wardle 2021).

In 2018, a similar scenario occurred in South Africa when a medical scheme retrieved R555 million through forensic audits, of which physiotherapists contributed R14 million (Smith 2019). However, data suggest that only 1% – 9% of audited healthcare professionals were found guilty of fraud, waste and abuse (FWA), with 1% – 6% cited for billing irregularities (Marais 2006). Despite this, 93% of the healthcare professionals were made to pay (Geldenhuys 2019).

Medical schemes are authorised by Section 59.3 of the *Medical Schemes Act* of 1998 to investigate inconsistencies in claim submission (Counsel of Medical Schemes 2021). However, they are not mandated to investigate FWA when the amount exceeds R100 000.00. Large amounts should instead be reported to the Health Professions Council of South Africa (HPCSA) and the South African Police Service (Health Professions Council of South Africa Corporate Affairs 2018; Meyer et al. 2024).

As medical schemes do not trust the police, HPCSA and the justice system, they do not report FWA. Instead, the scheme reclassifies the concerns as billing irregularities, keeping the HPCSA at bay (Marais 2006).

Artificial intelligence (AI) initiates the audit process by flagging irregular billing patterns of claims submitted to the scheme. Medical claims are submitted by patients, in cash practices or via electronic data interchange (EDI) systems, which have each code’s rules and regulations encoded into the system, preventing inconsistencies in claim submissions (Datamax 1995). Artificial intelligence can make errors that may result in false positives, leading to audits that may be unjustly conducted (Liu et al. 2022). If automation bias, misinterpretation of clinical practices and misuse of AI systems are used during audits, intentional or accidental harm may occur to practitioners and patients (Tamara et al. 2021).

One harmful ramification involves offset, where payments of new claims are withheld as repayment for alleged debts from older claims, without notifying the practitioner, and before establishing guilt (Deng et al. 2021). Offset, used as an apparent means of coercing an Admission of Debt (AOD) out of practitioners, creates an environment of intimidation and fear (Counsel of Medical Schemes, 2021).

Compounding these audits are the outdated tariffs available for billing in South Africa. The gazetted tariff codes have not been updated by the Competition Commission since 2006, owing to restrictions by the Competition Commission on ‘cohesive’ billing practices, and medical schemes do not accept new un-gazetted tariff codes (South African Society of Physiotherapy [SASP] 2016). Therefore, practitioners may be forced to use outdated codes to describe new evidence-based treatment modalities or not bill for these modalities.

The question that needs to be explored is how audits for billing irregularities affect South African physiotherapists, given the complex context. The question is important because of the negative ramifications reported internationally on medical professionals and patients (Faux et al. 2021). No study exploring this issue from the South African physiotherapy perspective could be found. This study investigated South African physiotherapists who had experienced forensic audits performed by medical scheme administrators in the private sector.

## Research methods and design

An analytical qualitative design explored and described the experiences of physiotherapists in South Africa regarding forensic audits by medical schemes. Semi-structured individual interviews and focus group discussions (FGDs) were conducted using an open-ended self-created interview guide (Kallio et al. 2016; Meyer et al. 2024).

### Participants

Fourteen private-practice physiotherapists, registered with the HPCSA and audited by medical schemes, were purposively recruited through purposive and snowball sampling. All audited physiotherapists accepted and gave written informed consent (Meyer et al. 2024).

### Data collection and analysis

Fourteen interviews were conducted: eleven personal interviews and FGDs comprising the group sizes of five, six and two participants, during three sessions (Meyer et al. 2024). The FGDs were used to expand on data gathered during individual interviews to elicit shared experiences that may not have emerged in the separate settings (Nyumba et al. 2018). Data were collected via Cisco Webex (https://signup.webex.com/sign-up), which recorded and automatically transcribed the interviews. Four trained interviewers conducted the sessions, three conducted three interviews each and one conducted four, with the lead author facilitating one FGD (Kallio et al. 2016; Meyer et al. 2024).

To thematically analyse the data, open coding was initially used, followed by axial coding, which identified the themes and subthemes (Medelyan 2019; Meyer et al. 2024). The main author conducted 11 rounds of data analysis, and the other authors corroborated the findings during ongoing conjoint meetings. Interviews were conducted as participants became available and in group discussions intermittently after every few individual interviews. Analysis of the first FGD resulted in saturation and was confirmed through recurring themes in subsequent interviews and FGDs. Bracketing was applied to minimise the bias and enhance the interpretive validity (Dörfler & Stierand 2021; Meyer et al. 2024). Reflection notes were made throughout using memoing and field notes and member checking further ensured data trustworthiness (Meyer et al. 2024; Nowell et al. 2017).

### Ethical considerations

Ethical clearance to conduct this study was obtained from the Faculty of Health Science Research Ethics Committee at the University of Pretoria (Reference no: 135/2022). All 14 physiotherapists who participated in this study gave written informed consent to participate in this study and for their data to be used in articles.

## Results

Participants (*n* = 14) had between 17 years and 55 years of experience in the physiotherapy field, working in special-interest practices. Of the practitioners, two had acquired a master’s degree and trained pre- and post-graduate physiotherapists. Twelve participants had both inpatient and outpatient practices, while two had exclusively outpatient practices. Most participants (*n* = 9) were solo practitioners, while five worked in a group practice.

Participants (*n* = 14) had all been audited by one major medical scheme. In contrast, one participant was additionally audited twice by a second medical scheme, resulting in a total of three audits. Eleven participants contacted the SASP for advice. One participant hired a forensic actuary, 10 paid for legal counsel and one physiotherapist had an advocate. Two physiotherapists met with the medical scheme without professional support. One physiotherapist futilely reached out to the HPCSA and the Council for Medical Schemes (CMS). In one practice, the legal fees amounted to as much as R500 000.00 (Meyer et al. 2024).

An AOD was signed by eight of the participants. At the time of the study, only two were found innocent, with one having retired, and they could not find fault with the second physiotherapist’s notes. Of the other participants (*n* = 4), one case was not finalised, two were still undergoing offset and one was blacklisted for being a cash practice. None of the participants had been investigated, nor had they been found guilty by the HPCSA or a court of law. Instead, they were forced to sign an AOD. Participants reported that the minimum amount audited was R54 000.00, and the maximum amount was R4.5 million in a group practice.

Six themes emerged from the analysis, supported by quotes from participants. Physiotherapists described feeling ‘unfairly persecuted, judged and penalised’ for being outliers, creating Theme One. Physiotherapists reported that auditors misunderstood their practices when they had a special interest that differed from a general physiotherapy practice. As a result, these physiotherapists felt unfairly accused of operating outside of conventional practice models. They further believed the penalties were disproportionate as auditors downplayed billing irregularities as being less serious than FWA, yet the financial penalties were more severe.

The second theme describes participants’ feelings of being overpowered when treated as if they were ‘criminal’. The forensic investigators implemented offsets, blacklisted participants, rejected evidence and ignored input from legal counsel.

In Theme Three, they felt unfairly treated and felt ‘naively entrapped between a rock and a hard place’ when they did not know what to expect. The participants had to continue paying for legal counsel, while medical schemes lacked transparency and abused their positional power.

In the fourth theme, participants described their distress with feeling ashamed, consumed mentally, emotionally drained, helpless and hopeless. In the fifth theme, physiotherapists felt hurt when stigmatised and labelled as guilty from the outset, resulting in harmful and detrimental effects such as financial losses and attrition of the physiotherapy profession.

### Theme One: Unfairly persecuted, judged and penalised

Participants reported feeling unfairly targeted and singled out, describing the audit process as ‘*unfairly conducted*’. Many felt they were subjected to ‘*a witch hunt*’, with one participant noting that their practice, which consisted of ‘*15 physios*’, with a ‘*specific orthopaedic range*’, was unfairly scrutinised. Others, like those in a ‘*paediatrics*’ and a ‘*neuro rehab practice*’, felt they were judged for being outliers. Others were audited for factors such as seeing high volumes of patients, whether in group practices or as sole practitioners who occasionally used locums for ad hoc support. A sole practitioner explained that they were accused of ‘*seeing too many patients as a sole practitioner, using locums*’. These experiences left participants feeling persecuted and misunderstood.

Participants felt that the investigators lacked the necessary clinical knowledge to accurately assess their practices, with one noting that the team consisted of ‘*actuarial scientists, accountants, attorneys, and maybe a forensic auditor*’. This perceived mismatch in expertise led to frustration, as physiotherapists believed the investigators failed to grasp the nuances of physiotherapy coding. As one participant pointed out, ‘*I have a fairly good working knowledge of physiotherapy coding*’, highlighting the gap in understanding between physiotherapists and investigators. Moreover, participants emphasised that they had ‘*used these codes forever*’, referring to gazetted tariff codes such as after hours, myofascial release, rehabilitation and complex evaluation, which have been unchanged since 2006. Physiotherapists were unaware that the scheme administrators scrutinised their application, which further exacerbated the sense of unfair judgement.

Participants felt that the investigators were driven by incentives that compromised their objectivity, with one physiotherapist, who had treated one of the investigators, reporting that the investigator told her she had ‘*won the incentive*’ for reaching the target and that ‘*their entire family went to the Maldives*’. This perceived conflict of interest contributed to a sense of mistrust, as participants believed that investigators would default to accusatory and derogatory positions, even in the face of doubt. Despite these concerns, participants were united in their stance, ‘*there is fraud, and the fraudsters need to be stopped*’, emphasising that any allegations ‘*need to be proven*’ through a fair and rigorous process.

Physiotherapists expressed that the processes were ‘*unfairly conducted and one-sided*’ as the scheme investigators repeatedly ‘*shifted the goalposts*’. These speak of misalignment between the penalty and the offence. When the understanding of codes was divergent, participants experienced being ‘*hauled over the coals*’. This came unexpectedly and made them feel that they are destined to ‘*burn in hell for the rest of your life*’, despite empathetic physiotherapists undercharging their patients: ‘*Most of the physios doing a good job, actually undercharge their patients*’.

Participants felt that penalties imposed were unjust, with one stating that ‘*the punishment given was completely out of proportion to the* [alleged] *“crime” committed*’. Specifically, physiotherapists were fined varying amounts, including ‘*ZAR1.2 million and ZAR4.5 million*’ for ‘*poor note taking*’, such as not recording the duration of each treatment session. Participants felt these charges were unwarranted, noting ‘*we’ve got the diary to prove it* [length of treatment]’. The code descriptions were also gazetted in the National Health Price Reference list (NHPRL), which specifies the required length of treatment time when charging rehabilitation. Additionally, the code descriptions were gazetted in the NHPRL. Despite this evidence, auditors rejected it, leaving physiotherapists feeling that the experience was ‘*quite upsetting*’. Furthermore, participants argued that the medical schemes’ decision to retain member savings, rather than refunding them, was unjust, ‘*because then they are actually making more money off their clients, which is fraudulent*’.

### Theme Two: Overpowered and oppressed

Physiotherapists felt that the forensic investigators overpowered them by their control over the payment process, describing experiences of being ‘*locked up in rooms, shouted at, forced to say that they were guilty*’. The forensic investigators were perceived as biased, with one participant stating she felt the investigators ‘*have a genuine hatred for physios*’, recalling a scheme investigator saying, ‘*You physio*[therapist]*s are all the same. You’re filthy, money-hungry, greedy people, and you all try and make as much money as you can; it’s disgusting*’.

This perceived animosity had a profound impact on participants, contributing to feelings of depression when their evidence was dismissed. Despite a comprehensive ‘*65-page forensic report*’ prepared by a forensic actuary in consultation with a physiotherapist, the investigators ‘*would not even look at it*’. Instead, participants were threatened with severe consequences, with the investigators stating that ‘*They will audit every single person on the list*’, and they ‘*will take back that money*’.

Physiotherapists felt overpowered by the scheme investigators’ control over payment processes. As one participant noted, ‘*I never received a cent from them*’, highlighting the investigators’ ability to withhold payments and use this as leverage to force participants to comply. This power imbalance was further exacerbated by the prolonged audit process, which was extended, ‘*five months*’ or longer, causing significant distress and limiting participants’ ability to operate their practices.

The investigators’ tactics were perceived as coercive, with participants being forced to either sign an AOD or face continued blockages on payments, effectively turning them into cash practices. Physiotherapists operating as cash-based practices were ‘*blacklisted*’ because scheme administrators could not use offset controls to manage claims. Despite contacting the SASP and seeking legal counsel, attempts to resolve the issue were ignored, and schemes compounded the situation by informing patients that ‘*they would no longer be reimbursing services*’. The physiotherapist was left with few options. One participant captured the sense of injustice, stating, ‘*They blacklisted me*’.

Furthermore, some participants were re-audited for the ‘*same codes*’, with one noting, ‘*You’re re-auditing me on the same codes that I’m not doing*’, demanding reversal of another 3 years of claim payments. One such physiotherapist protected his practice by making a deal. ‘*How about I pay you a certain amount of money a month, every month, until the SASP codes change*’.

### Theme Three: Naively entrapped between a rock and a hard place

Participants felt trapped in a state of uncertainty, with one noting, ‘*you don’t know what you’re in for*’. The financial burden of legal fees added to their stress, as ‘*everything that your lawyers finally sign*[s] *off … you pay for*’. This created a sense of constant apprehension, with participants feeling like ‘*you’ve always got this knife* [over your head]’ because of the perceived unfairness of the process. As one participant put it, ‘*It’s shocking, it’s unfair*’. The concentration of power in the hands of the investigators was particularly galling, with participants arguing that ‘*they cannot be judge, jury, and executioner*’.

The sense of entrapment was further exacerbated by the participants’ feeling that they were being set up, with the investigators ‘*set*[ting] *a trap*’ through divergent rules and contradictory behaviour. Participants felt powerless to defend themselves, describing the experience as facing ‘*this big bully*’ when ‘*you’re too small to fight*’. The schemes seemed to operate with impunity, like ‘*a law unto themselves*’, making it difficult for participants to understand their motivations, other than a perceived focus on extracting money. As one participant summed it up, ‘*we don’t really know what they’re after, except for money*’.

Participants felt deeply affected by what they perceived as false accusations from the medical scheme, leading to uncertainty about coding and billing practices, with one participant admitting, ‘*I just don’t bill for it*’ [A second condition]. This uncertainty stemmed from a conflict between their duty to prioritise patient care, as encapsulated in the phrase ‘*our patients come first*’, and the audit process invalidated their commitment to putting patients first.

### Theme Four: Distressed with a knife over your head

The distress participants experienced encroached on all aspects of their lives, ‘*it was consuming my everything*’. Their lives were evasively affected, including how they treated their patients, shopping and interactions with friends and family, draining them physically. ‘*My wife said … “You’ve got to start sleeping* [and] *eating because you’re just wasting away”*’. This was also expressed as ‘*I was in tears all the time*’. Participants reported having no energy, ‘*running on empty*’, as they felt isolated and without support, ‘*trying to get support*’, from organisations such as their professional society and the statutory board, while family, despite wanting to support, were powerless to do so: ‘*My dad … was helpless*’.

Participants felt consumed by hopelessness and helplessness, with one stating, ‘*You … have absolutely no hope*’. The emotional strain was profound, leaving participants feeling ‘*helpless, frustrated, very despondent, and very sad about my profession*’. The impact extended beyond the individual, affecting employers and family members, who sometimes required support, as one participant recalled, ‘*I had to calm her*, [the secretary] *down*’. The anxiety and stress were debilitating, with one participant describing a moment of intense distress. ‘*I was rocking in the bath with anxiety and stress*’.

The experience was marked by intense shame and embarrassment, with participants feeling ‘*like you are the worst human being on the planet*’, as you ‘*scramble to survive*’. The stigma associated with being audited made it difficult for participants to share their experiences with others, with one noting, ‘*there’s so much shame around it*’. The trauma was akin to post-traumatic stress disorder (PTSD), triggering physical reactions such as ‘*go*[ing] *into a cold sweat*’ when encountering reminders of the medical fund. The sense of violation and attack was overwhelming, with ‘*The word “blacklisted”* feeling like “*being shot in the heart”*’. For some, the experience was even more distressing than major life events, with one participant comparing it to watching a loved one die: ‘*Seven months watching my father die was easier than this experience*’. It was, in their words, ‘*the most horrible experience I went through*’.

The distress ultimately culminated in a sense of defeat, as participants exhausted their options and were forced to concede. For some, this resignation was marked by a sense of detachment, with one participant stating, ‘*I don’t care anymore, it’s only money*’. However, beneath the facade lay deep-seated anger about the perceived unfairness of the process and its significant financial costs. As one participant put it, ‘*That anger will never go away because the process has still appeared unfair*’. The experience also triggered profound self-doubt, with participants questioning their professional identity and purpose. For some, this introspection was intense, leading to ‘*a big questioning of my professionalism, my field, and even my billing system*’.

### Theme Five: Detrimental and hurtful

The participants experienced intense emotional distress, characterised by ‘*panic, high anxiety*’ that was all-consuming. For some, this was a new and debilitating experience: ‘*I mean, I never had depression or anxiety in my life*’. The anxiety permeated their daily thoughts, as they struggled to cope with the financial and professional implications: ‘*How are we going to do this? How are we going to afford this? Keep the practice going*?’ The emotional hurt was evident in their changed demeanour, ‘*you never smile anymore*’, and their work performance suffered as a result: ‘*You’re treating your patient. You’re not focusing on your work properly*’.

The participants also felt hurt by the perceived bias and stigma, being labelled as guilty from the outset. They described the experience of trying to reason with investigators who seemed unwilling to consider evidence: ‘*You’re giving the evidence to them as logically as you can, and they’re not even looking at it*’. This sense of futility was compounded by the fear of reputational damage, with participants anticipating that their names would be ‘*drag*[ged] *through the mud*’. As one participant warned, ‘*Trying to talk to someone who’s made up their mind, be prepared for them to try and slander your name*’.

Participants suffered significant financial losses, with one noting that ‘*The financial ramifications are probably the most pronounced*’. The burden of exorbitant legal fees, withheld payments and large debt penalties had a significant impact on their practices, particularly for sole practitioners. As one participant observed, ‘*We know many of our single colleagues; it was much harder. It almost destroyed their lives*’. The financial strain had a profound impact on participants’ well-being and trust in the medical scheme system, leaving them ‘*doubtful going forward* [concerning] *the viability of third-party funding*’. Some participants reported feeling drained of their passion and drive, stating ‘*Not having that love and drive I once had*’.

The participants warned that if the current trends continued, the consequences would be severe and far-reaching. They predicted that practitioners would experience extreme stress, leading to ‘*nervous breakdowns*’ and even impact their personal lives, resulting in ‘*miscarriages and getting divorced*’.

The well-being impact on physiotherapists was starkly illustrated by instances of severe health consequences. Although a direct causal link could not be established, ‘*one physio*[therapist] *had a stroke, and the other one had a miscarriage*’. The profession was facing attrition, with warnings that physiotherapists would ‘*emigrate and stop working in private practice*’. Moreover, patients were not immune to these challenges, sometimes facing dire outcomes when forced to pay out of pocket.

### Theme Six: Seeking remedies pre-emptively and preparedly

To mitigate the adverse effects of audits, improvements to billing education were proposed, including standardising billing courses and introducing them to undergraduate programmes. ‘*It needs to be a prerequisite somewhere*’, one participant emphasised. A simplified billing system was also advocated for, as the current system was seen as open to interpretation. ‘*The billing system is up for interpretation*’, highlighted the issue, exposing physiotherapists to billing irregularity audits and divergent understandings, with the medical schemes perceived as ‘*trying to find a new loophole to catch us*’.

Clearer guidelines and support from professional and statutory bodies were also seen as essential. The professional society was urged to take complete custodianship of the codes and standardise billing practices. ‘*The SASP needs to say these are the codes. You have to have this on your system*’, participants urged, seeking standardised billing practices. Although physiotherapists are first-line practitioners, they can only refer to themselves as having a special interest and not as a specialist, given the restriction of the HPCSA; ‘*I cannot say I’m a specialist in South Africa because of our rules*’. Furthermore, participants believed that business education should be integrated into undergraduate programmes to better equip physiotherapists with the knowledge of laws governing their practice, noting that ‘[running a business] *has got to feature a little bit more*’.

Ultimately, the physiotherapists felt that taking medical schemes to court could provide a platform for them to be heard and liberated, with one asserting, ‘*Take them to court and you’ll win*’.

## Discussion

This study explored the experiences of physiotherapists subjected to forensic audits by the medical funding schemes of South Africa. The findings reveal a seemingly unjust process caused by outdated regulatory codes, coercive practices and a lack of systemic transparency, resulting in distressing outcomes. These results add to the limited body of knowledge on audits conducted on South African medical professionals. This study further highlights the gaps in current auditing practices and regulations.

A key finding was that physiotherapists perceived the audit process as vindictive rather than beneficial. Like the findings of Faux et al. (2021), forensic investigators in this study were perceived as being guilty from the start, without considering alternative reasons for irregular billing patterns, and harsh penalties were imposed (Faux et al. 2021). These hostile auditing processes contradict the principle of procedural fairness, which requires fair treatment, transparency, impartiality and an opportunity to be heard (Garner 2019). Participants in this study reported that investigators received incentives, which, if substantiated, violates the Medical Schemes Act, as auditors may not be protecting the interests of members or ensuring fairness in their dealings because of potential biases (Counsel of Medical Schemes, 2021; South Africa 1998). Participants felt targeted and punished instead of supported and corrected.

Notably, penalties imposed by medical schemes for alleged ‘poor documentation’ vastly exceeded those set by the HPCSA for equivalent transgressions, raising concerns about fairness. In South Africa, when practitioners were accused of FWA and reported for investigation by the HPCSA, only R1000.00 to R3000.00 for poor note-keeping, which are gazetted penalties, were imposed (Hoffmann & Nortjé 2015). In this study, participants were coerced into signing an AOD, paying back penalties of up to R4.5 million for ‘alleged’ billing irregularities when forensic investigators deemed physiotherapists’ note-keeping not ‘good enough’. These differences suggest that the rules are not being applied consistently, raising concerns about whether the medical schemes follow the law or are held accountable for their actions (Peyton, Zigarmi & Fowler 2019). Additionally, when the ‘debt’ was repaid, most participants were re-audited despite the changes physiotherapists had implemented, highlighting the abuse of power.

Another concern was that audits were based on outdated billing codes, with schemes rejecting the original gazetted interpretations. Physiotherapists felt trapped, unable to defend themselves against alternative interpretations imposed by schemes. International research shows that rigid audits disconnected from clinical practice can harm healthcare quality and undermine professional autonomy (Van Fossen & Chang 2022). South African courts have ruled that regulations must be interpreted in their full context, not in isolation. This means understanding the broader purpose and intent behind the codes (Perumalsamy 2019; Rautenbach & Wallis 2019). When schemes ignore reasonable clinical justifications or interpret individual words without context, they may act unlawfully.

Furthermore, if these claims were accepted by EDI systems that have these rules encoded in the systems, then, as the Supreme Court stated, ‘*there is no sense in looking for the point in time when the debt is due, if the debt does not even exist*’ (*Standard Bank of South Africa Ltd v Miracle Mile Investments 67 (Pty) Ltd and Another* (187/2015) [2016] ZASCA 91; [2016] 3 All SA 487 (SCA); 2017 (1) SA 185 (SCA) (1 June 2016):15). Participants described how audits happened years after claims were processed, regardless of the contractual prescription and the *Medical Schemes Act*, which requires debt reports in a specific timeframe (South Africa 2010:21). Therefore, pursuing prescription claims undermines the power and authority of the law (*Trinity Asset Management (Pty) Limited v Grindstone Investments 132 (Pty) Limited* (CCCT248/16) [2017] ZACC 32; 2017 (12) BCLR 1562 (CC); 2018 (1) SA 94 (CC) (5 September 2017):7). Moreover, participants described how audits violated other existing laws when money paid from patients’ medical savings accounts was not returned, despite rulings from the *Genesis Medical Scheme v Registrar of Medical Schemes and another’* (CCT139/16) [2017] ZACC 16; 2017 (9) BCLR 1164 (CC); 2017 (6) SA 1 (CC) (6 June 2017) that funds should be protected as trust money.

Emotional distress was a huge theme, consistent with findings from other studies about mental health ramifications, such as moral injury and compassion fatigue (Bonsall 2020; Stoewen 2020). Participants reported anxiety, PTSD, burnout and feeling isolated and hopeless. The financial implications were severe, leading to profound distress, with the experience likened to the most challenging life events. Similar distress has been observed amongst practitioners, and it is further compounded when professional, statutory and societal organisations fail to provide adequate support (Dean, Talbot & Dean 2019; Van Fossen & Chang 2022).

A significant power imbalance exists between medical scheme administrators and individual physiotherapists who lack legal or institutional backing with jurisdiction over medical schemes. Participants felt intimidated and pressured to sign AOD agreements, reflecting the concerns described by Peyton et al. (2019), that organisations leverage power through a combination of force and reward, leading individuals to concede (Peyton et al. 2019). Additionally, Van Graan (2018) reported that innocent people will admit guilt when they experience techniques as oppressive (Van Graan 2018). The reported consequences included participants experiencing significant distress and profound disrespect towards the medical schemes, findings that align with research by Van Fossen and Chang (2022). To address this, participants recommended independent, unbiased mediators to ensure fair audit processes.

Importantly, participants suggested the system be improved through (1) updating and simplifying billing codes; (2) teaching legal and billing knowledge in undergraduate and postgraduate training; (3) creating support groups (e.g. WhatsApp communities) and (4) encouraging the professional bodies to take ownership of the custodianship of the codes.

These suggestions align with global best practices for making healthcare systems more transparent and supportive (Organisation for Economic Co-operation and Development [OECD] 2017; World Health Organization 2025). The findings emphasise the need for physiotherapists to have strong backing from civil societies to safeguard their interests and well-being. When people felt supported, their experience of isolation and mental well-being improved (Mushtaq et al. 2014).

Participants advocated for the HPCSA to recognise further qualifications and special-interest practices, while also upholding the status of first-line practitioners, similar to the approach taken in Australia (Bennett & Grant 2004; Goodwin et al. 2021). These changes would reduce mislabelling of physiotherapists with special interest practices as billing outliers. Physiotherapists require protection, particularly given the stark reality that in 2020, in South Africa, there were only 8053 registered physiotherapists responsible for treating 60 million people (Goodwin et al. 2021; Narain & Mathye 2023). The study’s findings can be cautiously applied to other healthcare professions, as the primary distinction lies in billing codes rather than the audit systems themselves.

A notable development since the completion of this study is the release of the long-awaited final report by an independent legal panel investigating the application of Section 59.3 of the *Medical Schemes Act* during forensic audits of medical practitioners (CMS 2025). The findings of this report corroborate many concerns raised by participants in this study, including retrospective application of audits, lack of transparency and the potential misuse of power by medical schemes. The release of this report is an important step towards institutional accountability and reform. However, the full implementation of its recommendations remains critical to ensure fair audit practices and to restore trust among healthcare providers.

Finally, investigations are needed to confirm the issues raised and replace outdated systems with fairer ones to reduce attrition. These organisations include the CMS, the Competition Commission, the Special Investigation Unit of South Africa and the Health Minister, who should determine whether current auditing practices meet ethical and legal standards.

### Strengths and limitations

The main strength was that the principal author was intrinsically involved and could fully understand participants’ experiences, which is sought after in creating depth in qualitative studies (Malka 2024). However, bracketing was applied to ensure the participants’ views were heard, not those of the principal author, ensuring the attention was on physiotherapists’ experiences (Dörfler & Stierand 2021). Interviews using FGDs and one on one and reflective journaling were used by the author to triangulate and capture detailed, personal stories.

However, there are several limitations. Firstly, participation was voluntary, and many physiotherapists were too scared to come forward. Those who participated may have biased the sample as they were more prepared to speak out. Secondly, the views of medical schemes were portrayed from the physiotherapists’ perspective as described in their interviews and may not be the medical schemes’ perspectives. Thirdly, all participants were audited based on one medical scheme, with only one participant audited using an additional scheme, adding to the contextual constraint of transferability.

## Conclusion

The study shows that current audit practices conducted by South African medical funding scheme administrators are harmful to physiotherapists. The recent release of the Section 59.3 final report validates the experiences reported in this study, highlighting the urgent need for systemic reform in audit practices, billing codes, legal protections and emotional support.

Professional bodies and government institutions must ensure that audits are conducted fairly, following the law and grounded in principles of care and justice. Physiotherapists must be supported to reduce attrition and mitigate the negative repercussions of forensic audits, which adversely affect the profession.
